# Analysis of Genome-Wide Alternative Splicing Profiling and Development of Potential Drugs in Lung Adenocarcinoma

**DOI:** 10.3389/fgene.2021.767259

**Published:** 2021-10-19

**Authors:** Jing Song, Jia Liu, Dekang Lv, Xuan Meng, Xiaodong Li

**Affiliations:** ^1^ Department of Respiratory Medicine, Qinzhou First People’s Hospital, The Tenth Affiliated Hospital of Guangxi Medical University, Qinzhou, China; ^2^ Department of Gynecology, Cancer Hospital of China Medical University, Dalian, China; ^3^ Institute of Cancer Stem Cell, Dalian Medical University, Dalian, China; ^4^ School of Medicine, Southern University of Science and Technology, Shenzhen, China

**Keywords:** lung adenocarcinoma, alternative splicing, splicing factor, prognosis, immune infiltration, small molecule drugs

## Abstract

Alternative splicing (AS) is significantly related to tumor development as well as a patient’s clinical characteristics. This study was designed to systematically analyze the survival-associated AS signatures in Lung adenocarcinoma (LUAD). Among 30,735 AS events in 9,635 genes, we found that there were 1,429 AS in 1,125 genes which were conspicuously related to the overall survival of LUAD patients. Then, according to the seven types of AS events, we established AS signatures and constructed a new combined prognostic model. The Kaplan-Meier curve results showed that seven types of AS signatures and the combined prognostic model could divide patients into distinct prognoses. The ROC curve shows that all eight AS signatures had powerful predictive properties with different AUCs ranging from 0.708 to 0.849. Additionally, the elevated risk scores were positively related to higher TNM stage and metastasis. Interestingly, AS events and splicing factors (SFs) network shed light on a meaningful connection between prognostic AS genes and corresponding SFs. Moreover, we found that the combined prognostic model signature has a higher predictive ability than the mRNA signature. Furthermore, tumors at high risk might evade immune recognition by decreasing the expression of antigen presentation genes. Finally, we predicted the three most significant small molecule drugs to inhibit LUAD. Among them, NVP-AUY922 had the lowest IC50 value and might become a potential drug to prolong a patient’s survival. In conclusion, our study established a potential prognostic signature for LUAD patients, revealed a splicing network between AS and SFs and possible immune escape mechanism, and provided several small-molecule drugs to inhibit tumorigenesis.

## Introduction

Lung cancer is a leading cause of cancer-related mortality worldwide (Chen et al., 2016; Siegel et al., 2021). Non-small cell lung cancer (NSCLC) is the most prevalent and heterogeneous subtype of lung cancer, including lung adenocarcinoma (LUAD) and lung squamous cell carcinoma (LUSC) (Chang et al., 2015). Generally, when LUAD and LUSC are in the same stage, the growth of LUAD is relatively slow with smaller masses. However, LUAD are more likely to start metastasis at the early stages. Although genomic data such as gene expression, DNA methylation, and copy number variation have been extensively studied in cancer, comprehensive and systematic analysis of alternative splicing is insufficient despite the fact that it has played a significant role in cancer (Ludwig and Weinstein, 2005; Koch et al., 2018; Han et al., 2019). Thus, it is necessary to further investigate the function of alternative splicing events on cancer recurrence and metastasis, especially in LUAD.

Alternative splicing (AS) is an extensive and sophisticated mechanism to increase the diversity of the proteome structurally and functionally (Climente-González et al., 2017). AS could exert a far-reaching influence on a protein’s biological characteristics by changing its stability, adding or deleting functional domains, controlling gene location, or regulating protein-protein interactions (Lee and Abdel-Wahab, 2016). Furthermore, it is a ubiquitous process and over 95% of genes undergo variable AS to produce a variety of transcripts (Feng et al., 2013). Normal alternative splicing could generate a multi-functional proteome to exert healthy cellular functions, while unusual alternative splicing can result in the occurrence and deterioration of cancers (Baralle and Giudice, 2017; Park et al., 2018). For instance, aberrant AS events could regulate the development and progression of a tumor by participating in several biological processes such as cell cycle progression, cell proliferation, and RNA processing. Accumulated studies have also highlighted that AS events have gradually become one of the hallmarks of carcinogenesis due to their unbalanced or incorrect expression (Ladomery, 2013). Thus, unusual AS events can be potential targets for cancer treatment.

The functions and roles of AS events in various cancers have been explored in a number of studies. For example, Bechara et al. (2013) demonstrated that NUMB alternative splicing regulated by RBM5, 6, and 10 could control lung cancer cell proliferation. Wan et al. (2019) verified that SRSF6 could mediate colorectal cancer progression by regulating alternative splicing. The alternative splicing of CCDC50 regulated by HnRNP A1 can result in clear cell renal cell carcinoma (ccRCC) tumorigenesis and development (Sun et al., 2020). However, few articles have systematically reported LUAD-specific AS events correlated with clinical characteristics.

Extensive dysregulated AS events in many types of cancers are easily programmed by many SFs, particularly the serine/arginine-rich (SR) and the heterogeneous nuclear ribonucleoproteins (hnRNPs) family. Different variate mature mRNAs were produced by SFs to assist spliceosome recognition and binding specific sequence of precursor mRNA (de Almeida and Carmo-Fonseca, 2012). The expression level of hnRNPs is different in many types of cancers, suggesting their extraordinary roles in tumorigenesis. Therefore, it is essential to depict an exhaustive regulatory network of SFs (Kędzierska and Piekiełko-Witkowska, 2017; Ratnadiwakara et al., 2018). Because the intimate correlation between AS and SFs was only superficially understood in view of their complexity, it is significant to investigate their potential prognostic performance, as well as their regulatory mechanism in LUAD.

The present study analyzed genome-wide LUAD-specific AS events using RNA-seq data in The Cancer Genome Atlas (TCGA) program, providing a systematically new understanding of the potential prognostic effects of AS events in LUAD. The purpose of this study was to clarify the roles of splice variants that could be considered as prognostic biomarkers in LUAD. Finally, the research uncovered interesting splicing networks in LUAD which could contribute to a better understanding of the fundamental mechanisms of LUAD.

## Material and Methods

### Alternative Splicing Events Data Collection

SpliceSeq data of TCGA-LUAD were downloaded from the TCGA database (https://tcga-data.nci.nih.gov/tcga/) (Tomczak et al., 2015). SpliceSeq tool, a java-based application, was usually used to unambiguously quantify the mRNA splicing levels of samples in TCGA. A novel value could be calculated by SpliceSeq based on seven types of AS events about each protein-coding gene provided from the Ensemble gene database (Ryan et al., 2012). For the following seven kinds of AS events, the Percent Spliced In (PSI) value was calculated, quantifying splicing event levels range from 0 to 1: Mutually Exclusive Exons (ME), Exon Skip (ES), Alternate Promoter (AP), Retained Intron (RI), Alternate Acceptor site (AA), Alternate Donor site (AD), and Alternate Terminator (AT). The schematic graph explaining these seven types of AS is shown in Supplementary Figure S1A.

### Establishment of the Prognostic Model

Patients’ clinical parameters of LUAD were downloaded and extracted from the TCGA database. In total, 444 LUAD patients were included in this analysis. Clinical information for these patients and the pathological details obtained from TCGA are provided in Supplementary Table S1. The PSI value of AS events in samples were collected and subjected to univariate Cox analysis. All the AS events screen for a *p* value <0.05, and these events were considered as candidate prognosis-related events. The “glmnet R″ software package was used to perform the least absolute shrinkage and selection operator (LASSO) analysis to filter out the most valuable and concise AS events in all AS events filtered in univariate Cox analysis (*p* < 0 .05). Afterward, the prognostic independence of the AS signature was constructed by multivariate Cox analysis. Then, based on the coefficient of each above AS event, each patients’ risk score could be calculated by the signature, respectively. Meanwhile, all patients were divided into distinct subgroups based on the median value of risk scores.

### Survival Analysis

The Kaplan-Meier curve was implemented to evaluate the differential survival status in both groups. The receiver operating characteristic curves (ROC) were conducted to detect both the sensitivity and specificity of prognostic signatures using the “survivalROC” R package (https://www.r-project.org/, v3.5.3).

### UpSet Plot and Splicing Factor Regulatory Network Establishment

We developed the Upset intersective plot, a more scalable visualizing diagram than Venn, which was used to explore the interactive sets of AS events. The “UpSet” R package was used to visualize their potential interrelationship. The expression data of the Splicing factors (SFs) was extracted from TCGA-LUAD mRNA-seq data. All SF genes were subjected to univariate Cox analysis when their *p* < 0.05. These SFs were considered the survival-associated splicing factors. The relationship closeness between SFs expression value and AS’s PSI value were calculated by the Spearman test. At the same time, the interaction network diagram of these SFs and prognosis-related AS events was illustrated using Cytoscape 3.7.0 (https://cytoscape.org/).

### Evaluation of Tumor Immune Cells Infiltrating

22 types of tumor-infiltrating immune cells were estimated using the CIBERSOFT algorithm, which characterized the cellular composition of complex tissues based on normalized gene expression profiles. The comparison of immune cell distribution between high and low-risk groups was made using the Mann-Whitney *U*-test.

### Identification of Potential Small Molecule Drugs

Potential drugs for the treatment of LUAD were selected using the Connectivity Map (CMap) database (https://clue.io/). We uploaded differentially expressed genes between high and low-risk groups into the CMap database for genomic enrichment analysis. We screened the small drug molecules with an enrichment score of <90, and obtained 3D structure through PubChem database (http://www.pubchem.ncbi.nlm.gov), a public repository of small molecules in properties. The IC50 value of these small molecule drugs was provided by the GDSC database (https://www.cancerrxgene.org/).

## Results

### Overview of AS Events in TCGA-LUAD

Comprehensive AS events were examined in a cohort of 444 TCGA-LUAD patients (Supplementary Figure S1A).A total of 30,735 AS events from 9,635 genes were detected, including 11,768 ES events in 5,467 genes, 6,129 AP events in 3,424 genes, 5,782 AT events in 3,372 genes, 2,605 AA events in 1,961 genes, 2,199 AD events in 1,659 genes, 2,103 RI events in 1,461 genes, 149 ME events in 146 genes (Supplementary Figure S1B). In TCGA-LUAD, ES events were the most common AS events, accounting for approximately just over one-third of all events, followed by the number of AP and AT events, while the number of ME events was the least. Remarkably, the number of AS events went far beyond those of their corresponding mRNAs. Furthermore, a subset of overlapping AS events in the seven types of AS in LUAD was displayed by the UpSet plot diagram (Supplementary Figure S1C).

### Identification of Prognosis-Related AS Events in LUAD

**FIGURE 1:**
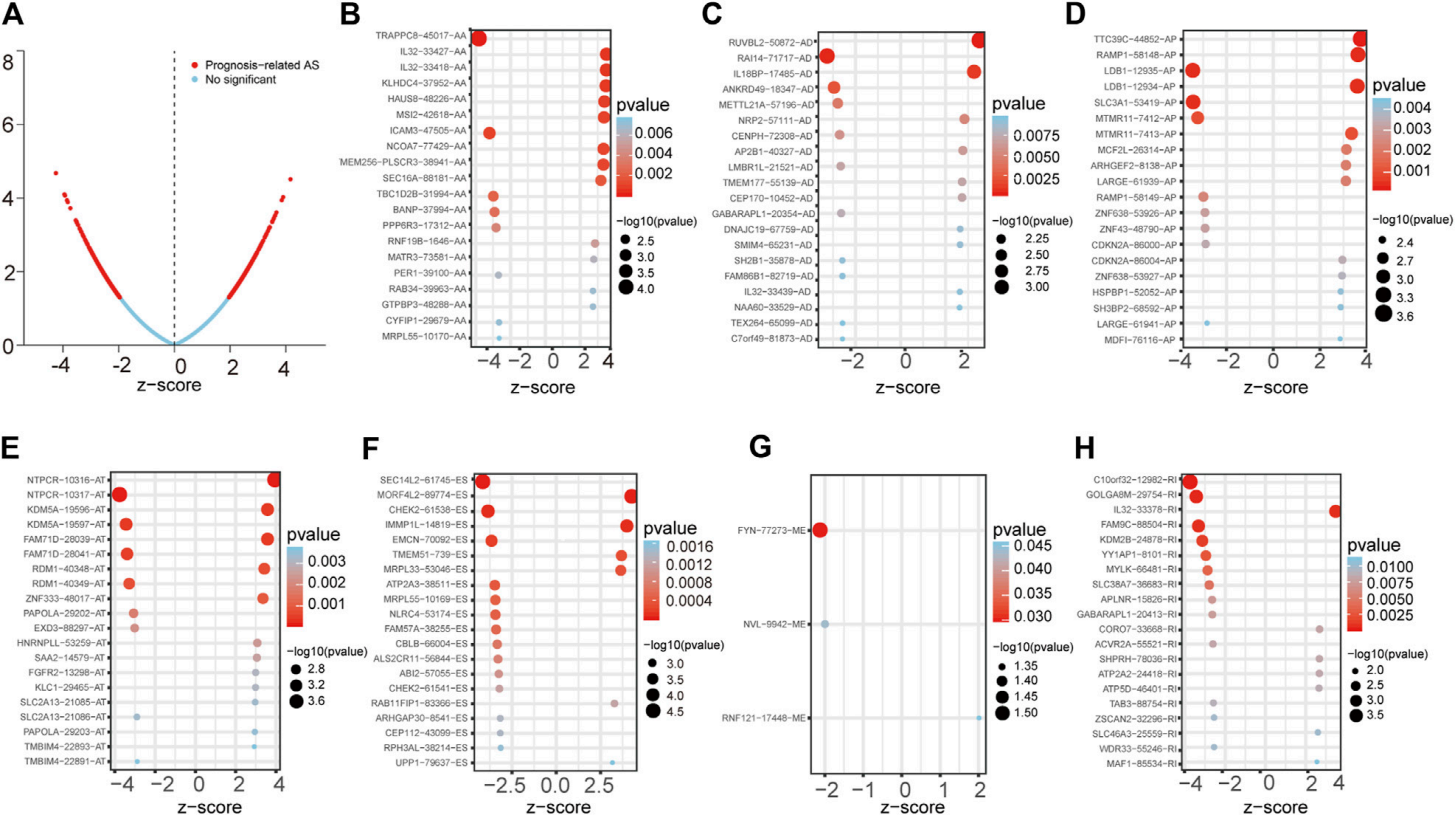
Forest plots analyses of survival-associated AS events. **(A)** Volcano plot depicting the *p* values from univariate Cox analysis of the 30,735 AS events. **(B–H)** Forest plots of z-score of the top 20 significantly survival-related AS events for seven splicing types (ME only three events).

First, we conducted a univariate Cox analysis based on the 30,735 AS events related to 444 patients to appraise the relationship between AS events and overall survival (OS) status in LUAD. Consequently, 1,429 AS events and corresponding 1,125 genes were conspicuously related to the overall survival of LUAD patients (Figure 1A). Figures 1B–H showed the top 20 most important AS events related to OS among these seven types of AS events. Interestingly, some special survival-associated AS genes contain multiple types of AS events. For example, AD, AT, AA, RI, and ES of C1orf159 and AP, AA, RI, AD, and ES of MRPL55 were all related to OS of LUAD patients.

### Establishment of Prognostic AS Signatures

**FIGURE 2:**
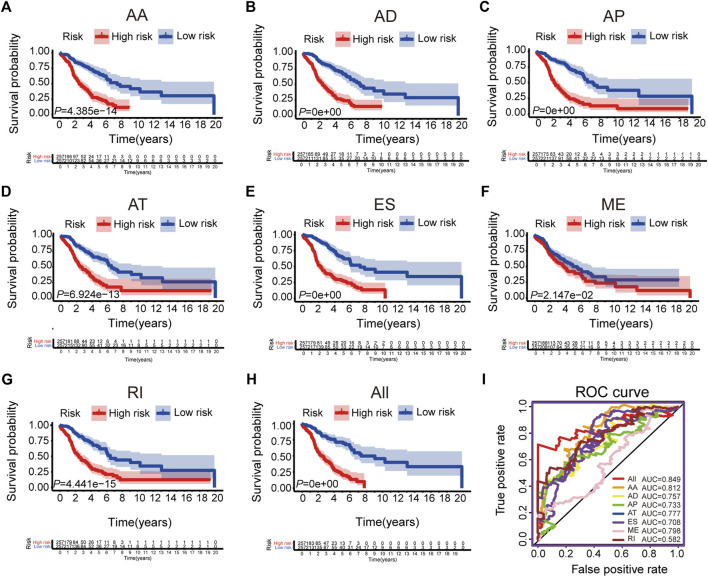
The Kaplan-Meier curves and ROC curves of prognostic AS models. **(A–G)** The Kaplan-Meier plots of seven types of AS events, respectively. **(H)** The Kaplan-Meier plots of combined prognostic model. **(I)** The ROC curves for overall survival of seven types of AS events and combined prognostic model.

We selected the significant prognostic associated AS events as candidates by univariate Cox analysis, aiming to further screen out the most significant AS events related to patient prognosis by LASSO Cox regression analysis (Supplementary Figure S2). Furthermore, several prediction signatures based on these prognostic associated AS events were constructed by multivariate Cox analyses. Eventually, a combined prognostic model was built, integrated from different types of AS events (Supplementary Table S2). The median value of risk scores was considered as the cutoff criteria for dividing patients into a high-risk group and a low-risk group. The Kaplan–Meier curves shown in Figures 2A–H, showed that LUAD patients in the high-risk group had appreciably shorter OS than patients in the low-risk group, demonstrated that these AS signatures could be powerful biomarkers to distinguish patient prognosis. The combined prognostic signature showed better predictive properties than the single type of AS events (Figure 2H). Then, the ROC curve was performed to appraise the prognostic efficiency of prognostic AS models. The results show that all signatures had a robust predictive property with AUC values from 0.708 to 0.849, except the ME signature (AUC = 0.582, Figure 2I). Conceivably, the combined model contains different types of AS events that had the highest efficiency (AUC = 0.849). The distribution diagram of patients’ risk score, survival status, and expression profiles of related AS model events are shown in Supplementary Figures S3A–H.

### Validation of Combined AS Signature

To confirm the prognostic value of our combined AS signature, we randomly selected 50% of patients in all LUAD patients as a testing cohort and performed Kaplan-Meier cure and ROC analysis (Supplementary Figure S4). The results were consistent with the above results and demonstrated the combined AS signature had a robust predictive ability (AUC = 0.856).

### AS Signatures Are Independent Factors for Other Clinical Characteristics

**FIGURE 3:**
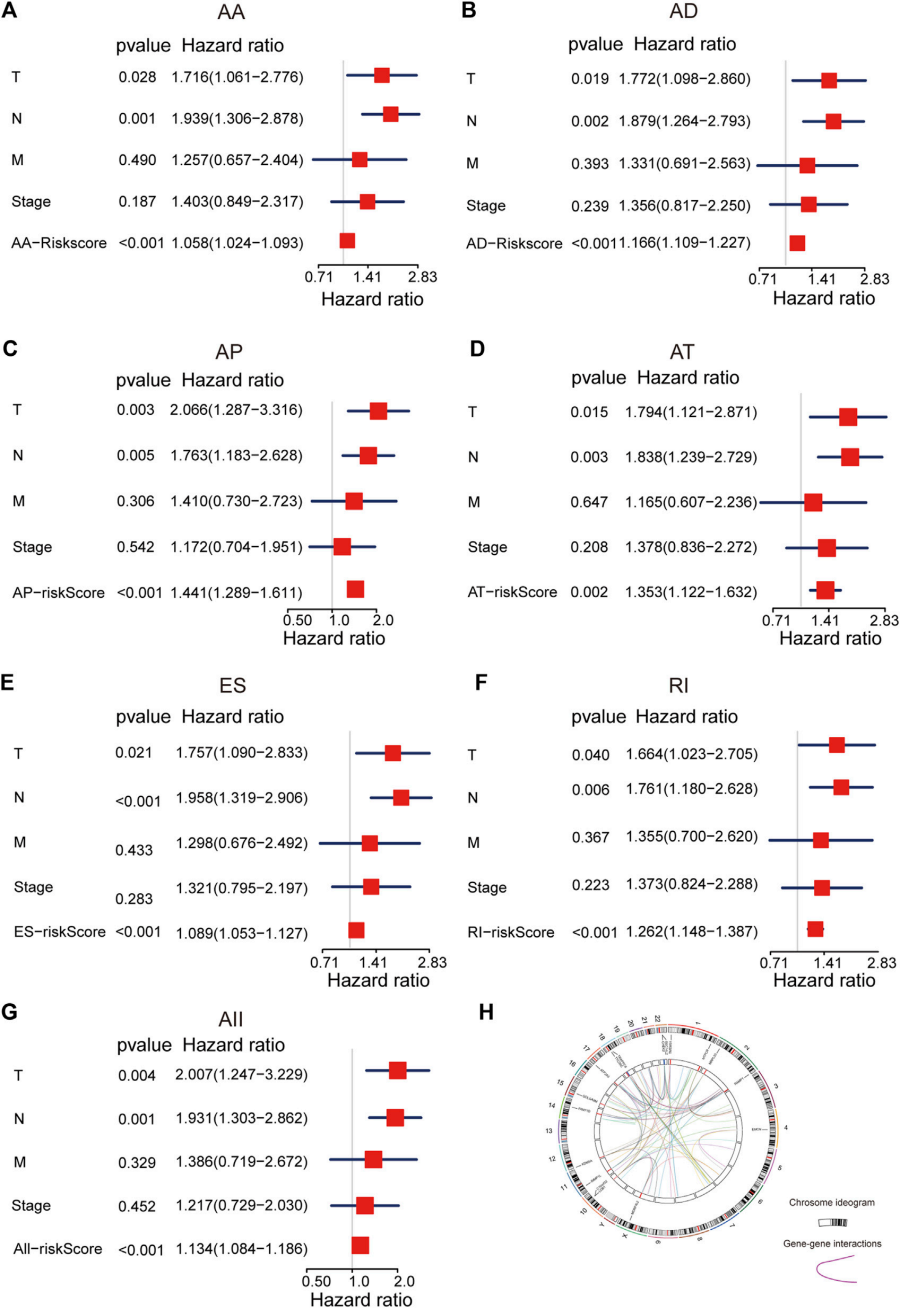
The multivariate Cox analysis of AS signatures and clinical characteristics. **(A–G)** Forest plots of hazard ratios of risk scores and clinical characteristics from multivariate Cox analyses. **(H)** Circos plots of the detail of AS events and its interacting genes in chromosome.

To explore the predicted performance of AS signatures and other clinical characteristics of survival, univariate and multivariate Cox analyses were performed to find out if these AS signatures were independent prognostic factors for LUAD patients. The univariate Cox analysis showed that almost all risk score signatures (except ME-risk score signature), TNM stage, T, N, and M stage were remarkably related to LUAD patients’ overall survival (Supplementary Figure S5). Furthermore, multivariate Cox analysis results show that most risk score signature, T and N stage still have a predictive ability when all univariate significant factors are considered together, suggesting that risk score, T and N stage were unassisted risk elements (Figures 3A–G). Taken together, all these results demonstrated that AS signatures exhibit powerful predictive performance in LUAD patients. In addition, circos plots were depicted to display the details of AS events and their interacting genes in the chromosome (Figure 3H).

### Network of Prognosis-Related AS Events and SFs

**FIGURE 4:**
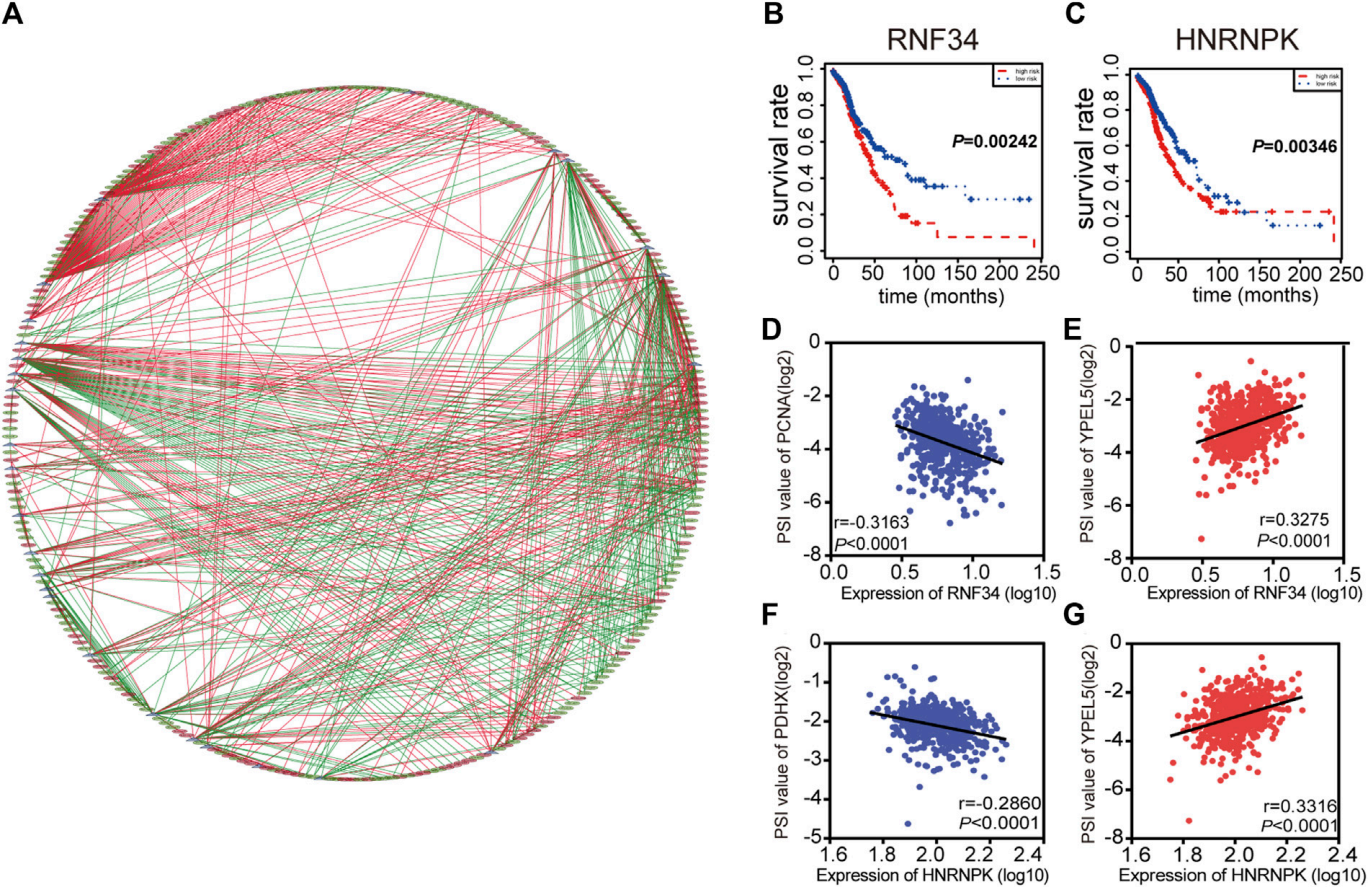
Splicing correlation network in LUAD. **(A)** Correlation network between expression of survival Splicing factors and PSI values of AS genes generated using Cytoscape. Purple dots were survival associated splicing factors. Green/Red dots were favorable/adverse AS events. Red/Green lines represent positive/negative correlations between substances. **(B–C)** Kaplan-Meier curve of splicing factors RNF34 and HNRNPK. **(D–G)** Representative dot plots of correlations between expression of splicing factors and PSI values of AS events.

Interestingly, extensive dysregulated AS events in many types of cancers are easily programmed by some specific SFs. Thus, an interesting issue is whether several key SFs could regulate these prognosis-associated AS events in LUAD. To determine those specific SFs which had a close connection with prognosis-associated AS events in LUAD, univariate Cox analysis of all SFs were performed based on the gene expression value of LUAD patients. The results showed that there were 30 SFs related to the OS of LUAD patients (Supplementary Table S3). Furthermore, correlations between SFs and prognostic AS events were tested by Spearman’s test (Figure 4A). In the correlation networks, 27 SFs (purple dots) were related to 248 prognosis-associated AS events, involving 141 favorable AS events (green dots) and 107 adverse AS events (red dots). Intriguingly, there was a positive relationship (red lines) between most of the poor survival-related AS events (red dots) and SFs (purple dots), while there was a negative correlation (green lines) between most of the favorable survival-related AS events (green dots) and SFs. For example, SFs RNF34 and HNRNPK were related to worse survival of LUAD patients (Figures 4B,C). ES events of YPEL5 were an adverse factor, while the AP events of PCNA and AP events of PDHX were related to a favorable prognosis. The relationship between RNF34 and the AP of PCNA or ES of YPEL5 were shown in dot plots, implicating the high expression of RNF34 was negatively related to adverse prognosis (Figures 4D,E). Similarly, the relationship between HNRNPK and the AP of PDHX or ES of YPEL5 were shown in dot plots (Figures 4F,G), illustrating that the high expression of HNRNPK was positively related to poor prognosis.

### The AS Signature has Better Predictive Ability Than the mRNA Signature

**FIGURE 5:**
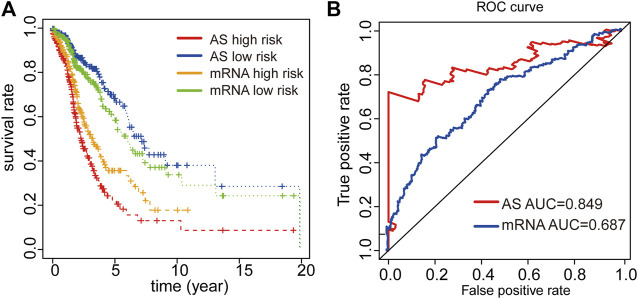
Comparison of Kaplan-Meier and time-dependent ROC analysis of our AS signature with mRNA signature. **(A)** Comparison of Kaplan-Meier analysis of our AS signature and mRNA signature. **(B)** Comparison of ROC analysis of the sensitivity and specificity of our AS signature and mRNA signature.

We finally constructed an mRNA signature for LUAD patients by subjecting differentially expressed mRNAs in LUAD to univariate and multivariate Cox analysis: mRNA risk score = (0.1674*XPR1) + (0.0529*MMP1) + (-0.2108*MAP3K8) + (0.0882*RHOV) + (0.1099*CDH17) + (0.1488*UCA1). Then, Kaplan-Meier and ROC curves were implemented to contrast prognostic ability between AS signature and mRNA signature. Both results from Kaplan-Meier and ROC analyses illustrated that AS signature had significantly better survival and higher ROC than mRNA signature (Figures 5A,B). These data demonstrated that the predictive power of AS signature is greater than the mRNA signature. In general, AS signature could be used as a superior indicator to predict the prognosis of LUAD patients.

### Tumor Microenvironment Cell Infiltration Characteristics Related to Drug Resistance

**FIGURE 6:**
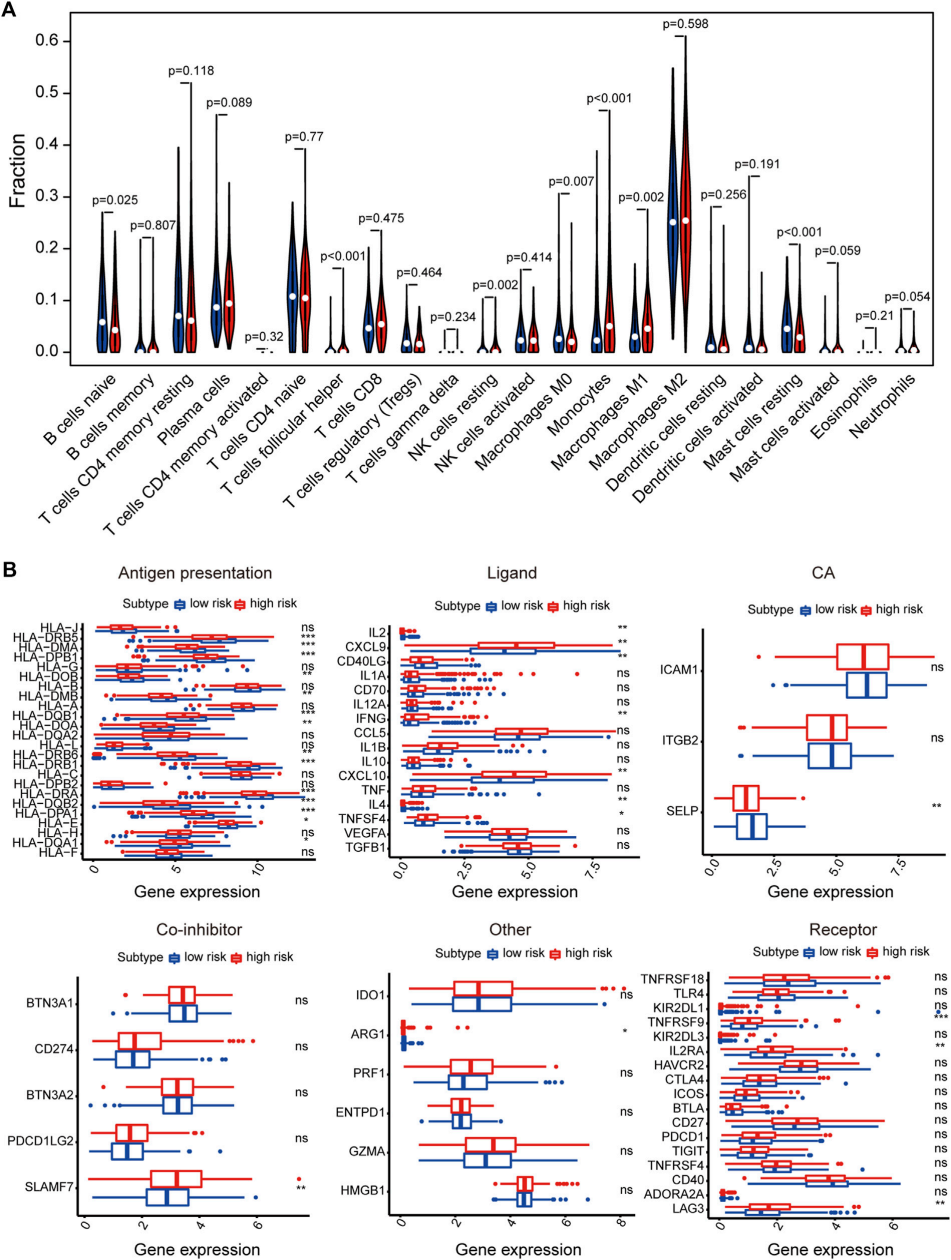
The relationship between signature and immune-related features. **(A)** The violin plot of the 22 immune cell proportions between high and low-risk groups. **(B)** Effect of signature on the expression of different immunomodulators. *p < 0.05, **p < 0.01, ***p < 0.001, ****p < 0.0001.

We also analyzed tumor microenvironment cell infiltration of signature. We calculated the median absolute score that CIBERSORT gave for 22 cell types in two groups. The results showed that the fraction of T cells follicular helper, NK cells resting, Monocytes, and Macrophages M1 was significantly higher in high-risk than that in the low-risk group. However, B cells naive, Macrophages M0 and Mast cells resting were remarkably higher in the low-risk than in high-risk group (Figure 6A). Furthermore, the results showed that the expression of some immunomodulator agonists was correlated with a risk score. We found that some immune ligands and receptors were significantly higher in the high-risk group, while antigen presentation was mainly higher in the low-risk group (Figure 6B).

### Related Small Molecule Drugs Screening

**FIGURE 7:**
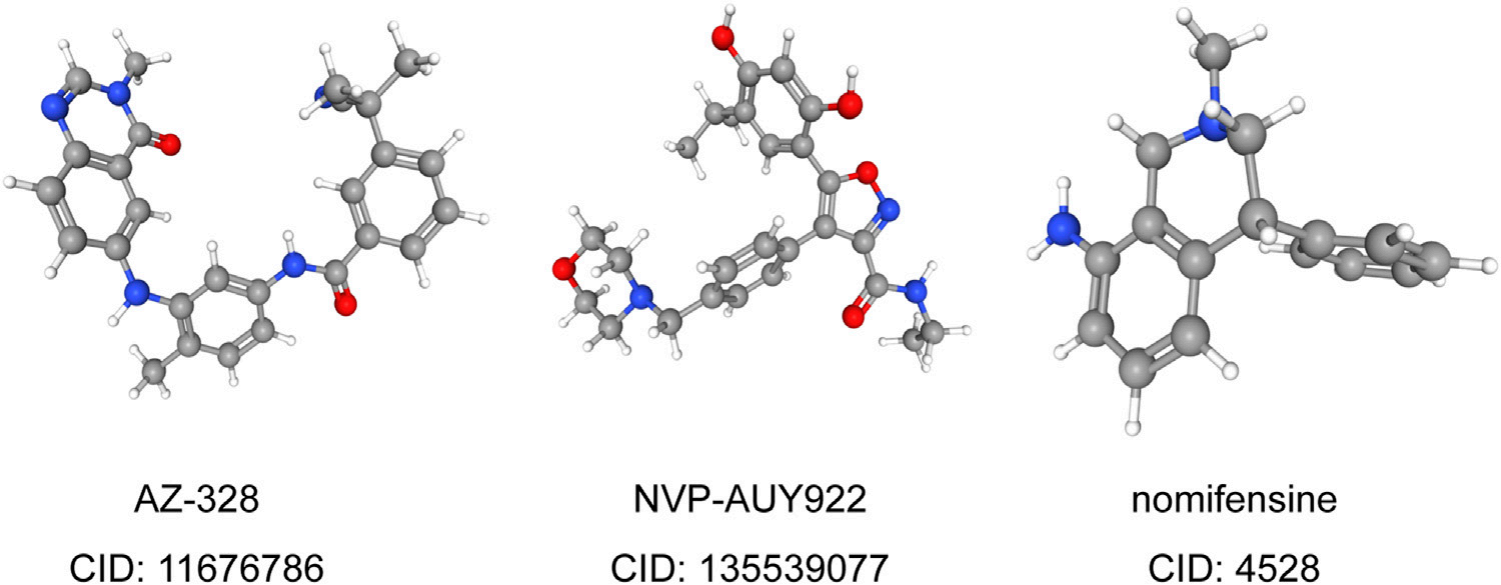
3D conformer of most significant small molecule drugs.

To predict small molecule drugs that can inhibit the resistance of LUAD patients, DEGs of high-risk and low-risk groups were assigned into up-regulated and down-regulated groups. Then we matched it to a small molecule drug in the CMap database. Finally, we selected the three most important small molecular compounds, including AZ-628 (score = −95.17), NVP-AUY922 (score = −91.77) and Nomifensine (score = −90.86). The 3D structure of these three small molecular compounds was downloaded from the PubChem database (Figure 7). These small molecules might potentially improve the outcomes of LUAD patients and provide recommendations for the selection of LUAD-targeted drugs, while the specific mechanism and effectiveness need to be further studied. We also explored the IC50 values of these small molecule drugs in LUAD cells through the GDSC database. The results showed that NVP-AUY622 (IC50 = 0.004–10.222 μM) had lower IC50 values than AZ-628 (IC50 = 0.050–46.073 μM), indicating that NVP-AUY622 has a stronger effect in LUAD cells (nomifensine not provided). These results demonstrated that NVP-AUY922 might become a novel drug to improve the survival of LUAD patients.

## Discussion

Alternative splicing of pre-mRNA is a ubiquitous and flexible process, which could provide the opportunity for cells to generate different or even the opposite functions of protein isoforms from a single gene. Furthermore, this flexibility is usually used by tumor cells to generate proteins that facilitate growth and progression (David and Manley, 2010). Changes in AS might affect all aspects of tumor biology, including metastasis, invasion, metabolism, and apoptosis, as well as angiogenesis (Blencowe, 2006; Moore et al., 2010; Coomer et al., 2019). For example, *David et al* suggested that regulating AS events can contribute to tumor cell proliferation (David et al., 2010). KLF6-SV1 (splice variant 1) exerts an essential role in the development and progression of cancer (Narla et al., 2005; DiFeo et al., 2009). All in all, it is necessary to further investigate not only mRNA expression levels but also some splice variants.

In recent decades, accumulating studies have shown that AS events affect proliferation, migration, radioresistance, and so on (Yae et al., 2012; Sheng et al., 2018; Wang et al., 2020a). For example, Sheng et al. (2018) found that SRSF1 is involved in radioresistance in lung cancer cells through modulating the aberrant splicing of PTPMT1. In addition, QKI-5 could inhibit cell proliferation and the migration of lung cancer via regulating the splicing of ADD3 exon 14 (Wang et al., 2020a). However, prognosis-related AS events in LUAD remain mostly unstudied, especially in patients with metastasis.

SpliceSeq, a novel convenient exploited analysis pipeline (integration tool), was used to detect AS events, which could help analyze complex or low-frequency AS events (Ryan et al., 2012). Here, we systematically and comprehensively analyzed a total of 30,735 AS events in a TCGA-LUAD cohort and identified 1,429 prognosis-associated AS events in 1,125 genes. Our results indicated that a combined prognostic signature containing several kinds of AS events had excellent performance in survival prediction. More importantly, we revealed that some AS events were associated with the metastasis of LUAD patients, which might be a meaningful discovery in further exploring the mechanisms of LUAD metastasis. In a previous study, Tyler et al. detected some splicing variants and found that CHEK2 is a prominent suppressor gene in cancer (Landrith et al., 2020). KDM5A could promote SCLC proliferation and metastasis *in vivo* by repressing the NOTCH signaling pathway (Oser et al., 2019). These results have been verified in our analysis.

As an increasing number of studies have demonstrated that AS events are modulated by some pivotal SFs (Cieply and Carstens, 2015; Song et al., 2018), the analysis of SFs was also underlined in this study. Intriguingly, the splicing correlation network suggested that there was a positive relationship between most poor survival-related AS events and SFs, while there was a negative correlation between most favorable survival-related AS events and SFs. For example, HNRNPK have adverse factors in LUAD and were found to be positively associated with ES of YPEL5, which was also considered an adverse factor. Moreover, HNRNPK has been reported and could promote metastasis in lung cancer (Li et al., 2018; Li et al., 2019). It is fair to say that poor-survival SFs might facilitate the occurrence of adverse prognostic AS events. Nevertheless, it is necessary to further explore the more specific regulatory mechanisms of AS-SF networks.

In the analysis of the correlation between risk score and immune cells, we found that T cells follicular helper, NK cells resting, Monocytes and Macrophages M1 were mainly enriched high-risk group, while B cells naive, Macrophages M0 and Mast cells resting were enriched in the low-risk group. Jhunjhunwala et al. (2021) that found tumors can down-regulate the expression of HLA-1 by interfering with antigen processing and presentation mechanisms to achieve tumor evasion immune recognition. In our study, most of the antigen presentation genes were a low expression in a high-risk group, which might promote tumor immune escape.

More importantly, we identified the three most significant small molecule drugs, including AZ-628, NVP-AUY922, and Nomifensine, that might improve the survival of LUAD patients, In particular, NVP-AUY622 has the lowest IC50 value in LUAD cell lines. Among these three drugs, AZ-628, an RAF Kinase inhibitor, can reverse cancer multidrug resistance (MDR) by mediating ATP-Binding Cassette Transporter G2 (ABCG2) (Wang et al., 2020b). NVP-AUY922 has been found to have potent anti-tumor activity and can inhibit tumor growth, including NSCLC, breast cancer, colorectal cancer, and so on (Jensen et al., 2008; Garon et al., 2013; Lee et al., 2015). In colorectal cancer cells, HSP90 inhibitor NVP-AUY922 can suppress the JAK2-STAT3-Mcl-1 signaling pathway to enhance TRAIL-induced apoptosis (Lee et al., 2015). At present, as an antidepressant, Nomifensine is mainly used to diagnose and test hyperprolactinemia (Müller et al., 1978). However, the anti-tumor effect of Nomifensine needs further confirmation.

In summary, this study indicated the prognostic value of some AS events in LUAD and patients with metastasis, which could modulate some key SFs. These results further understanding of the interaction between AS and SFs in LUAD, indicating that the systematic analysis of AS signatures in LUAD might contribute multiple potential biomarkers and the underlying mechanisms of LUAD metastasis. In addition, we provided three small molecule drugs for treatment selection of LUAD.

## Data Availability

The original contributions presented in the study are included in the article/Supplementary Material, further inquiries can be directed to the corresponding author.
