# Supplementary material for: Analysis of Genome-Wide Alternative Splicing Profiling and Development of Potential Drugs in Lung Adenocarcinoma
Source: Front Genet. 2021 Oct 19;12:767259. doi: 10.3389/fgene.2021.767259 (PMC8560713; doi:10.3389/fgene.2021.767259)
Supplement: Supplementary file 1 [file Table1.DOCX]

Table 1. Three most significant small molecule drugs

| Name | Score | CID | Description | Rank |
| --- | --- | --- | --- | --- |
| AZ-628 | -95.17 | 11676786 | RAF inhibitor | 1 |
| NVP-AUY922 | -91.77 | 135539077 | HSP inhibitor | 2 |
| nomifensine | -90.86 | 4528 | Dopamine uptake inhibitor | 3 |
