# Supplementary figures and images for: Analysis of Genome-Wide Alternative Splicing Profiling and Development of Potential Drugs in Lung Adenocarcinoma
Source: Front Genet. 2021 Oct 19;12:767259. doi: 10.3389/fgene.2021.767259 (PMC8560713; doi:10.3389/fgene.2021.767259)

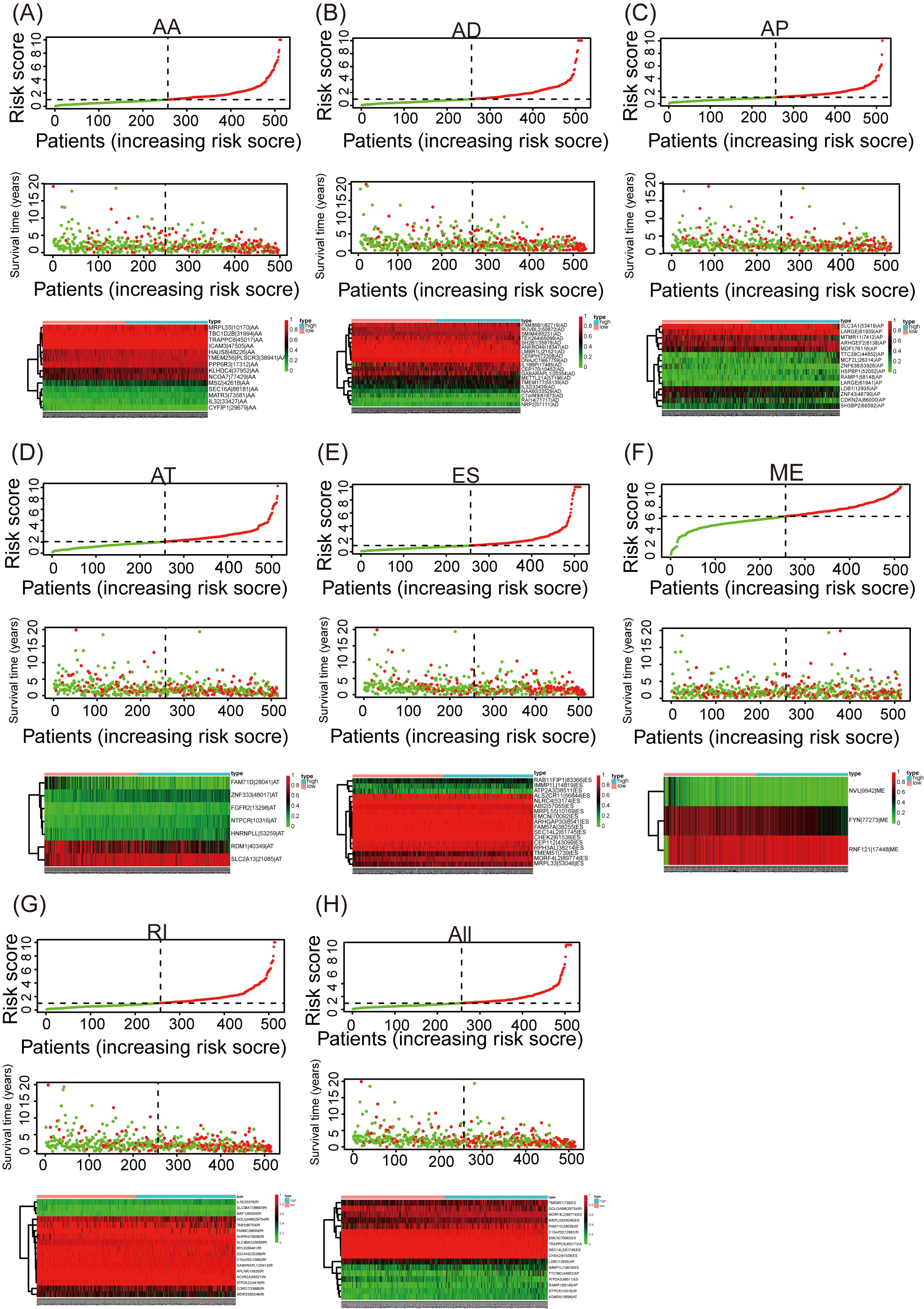

Supplement: Supplementary file 3 [file Image3.TIF]

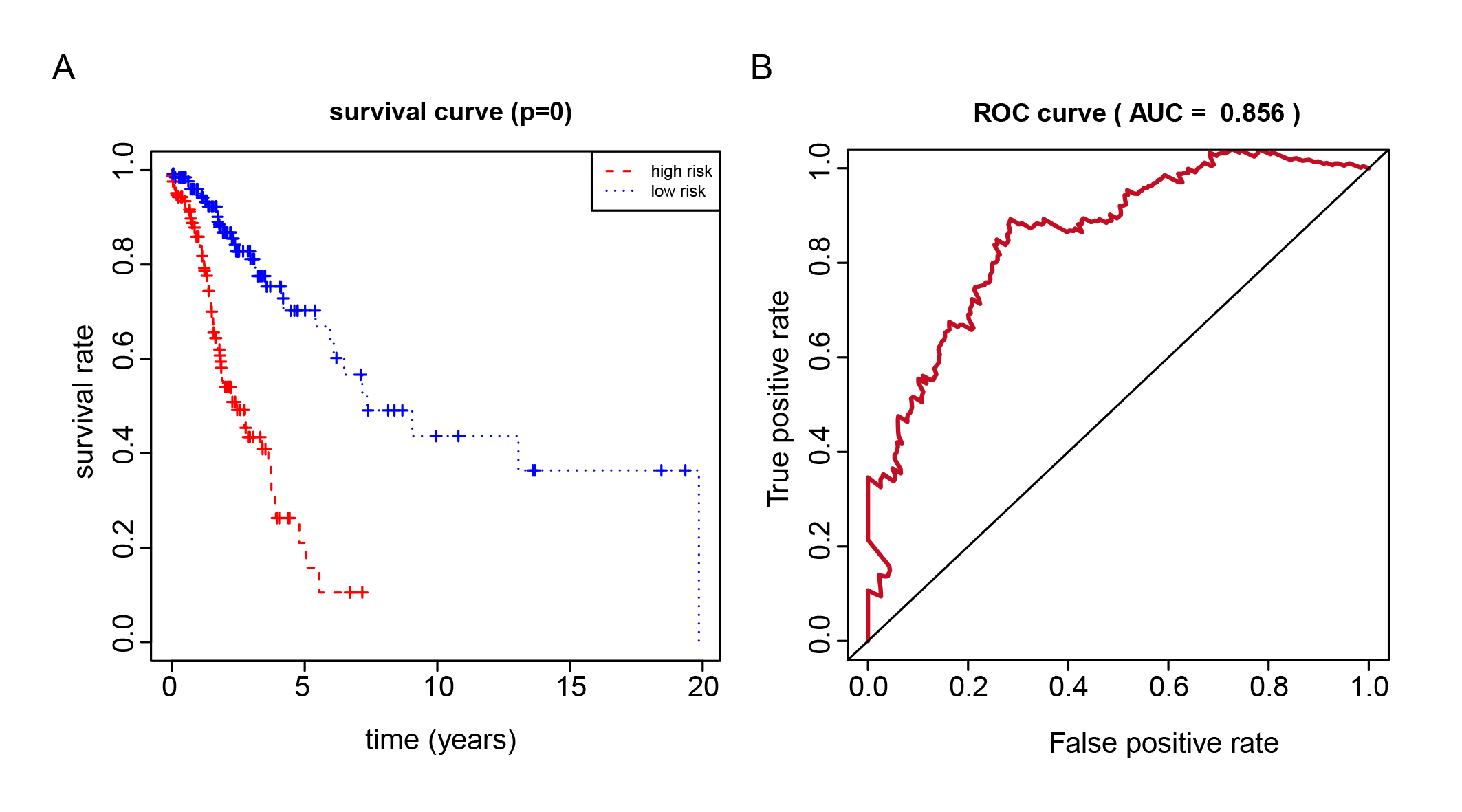

Supplement: Supplementary file 4 [file Image4.TIF]

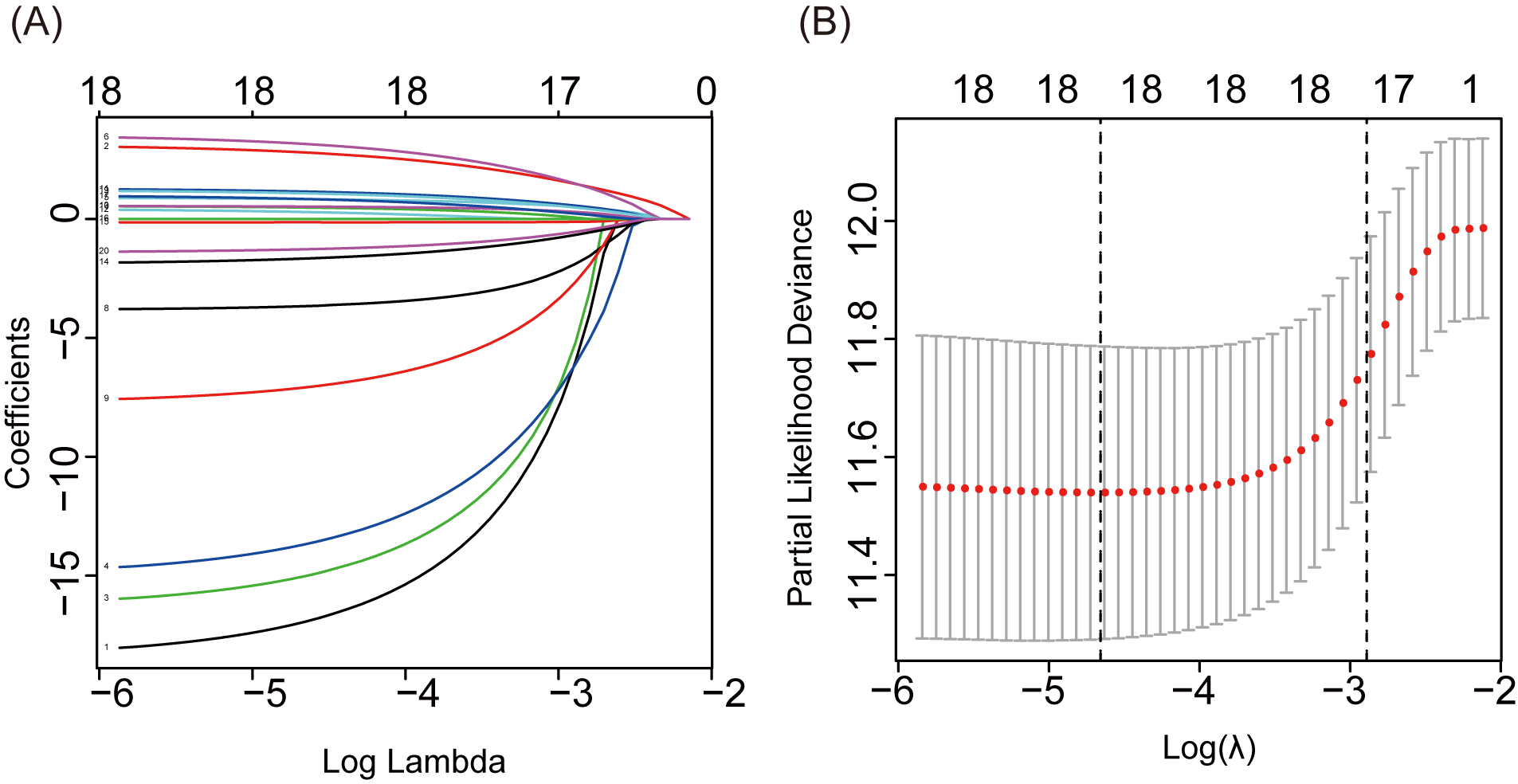

Supplement: Supplementary file 5 [file Image2.TIF]

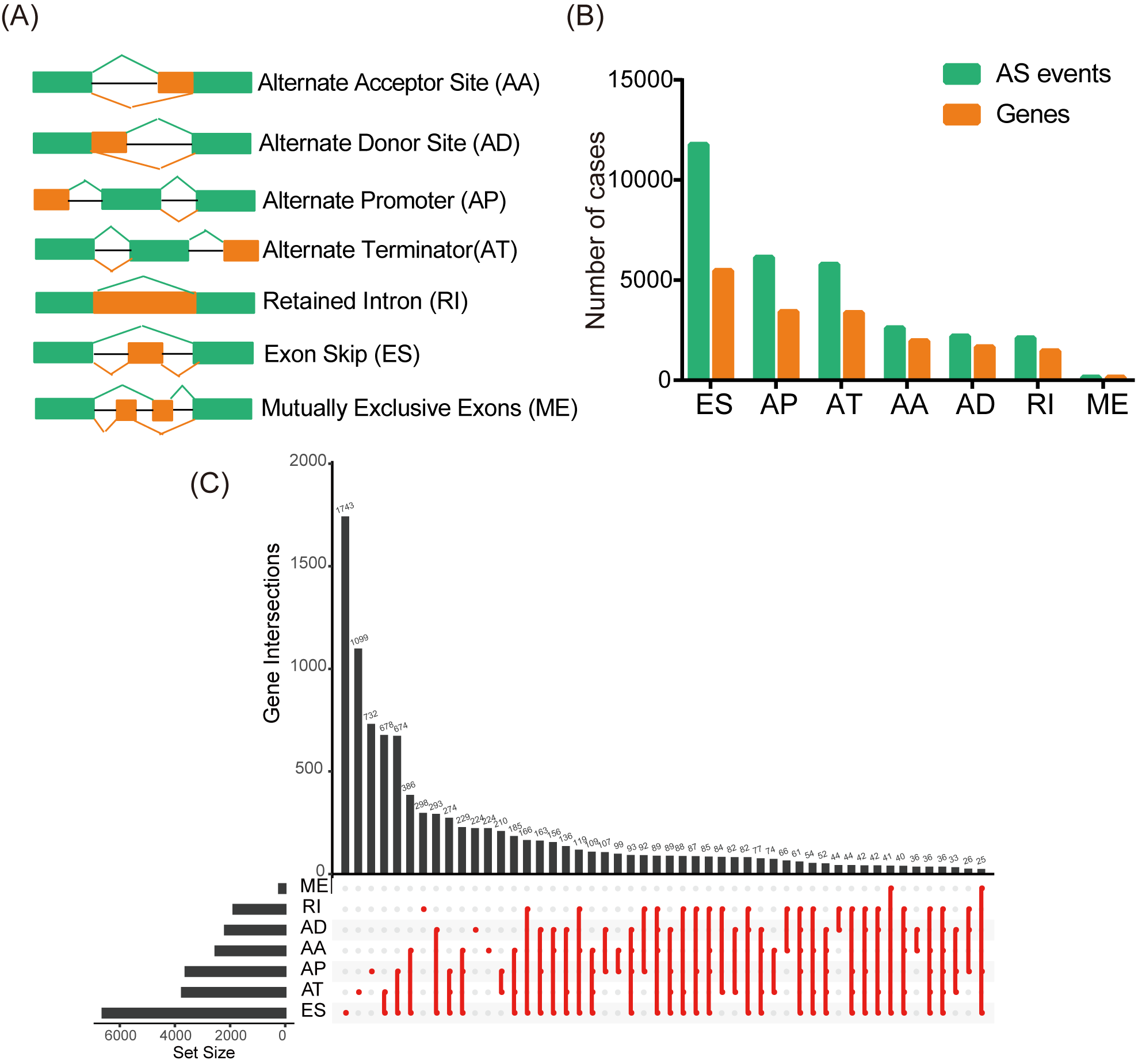

Supplement: Supplementary file 6 [file Image1.TIF]

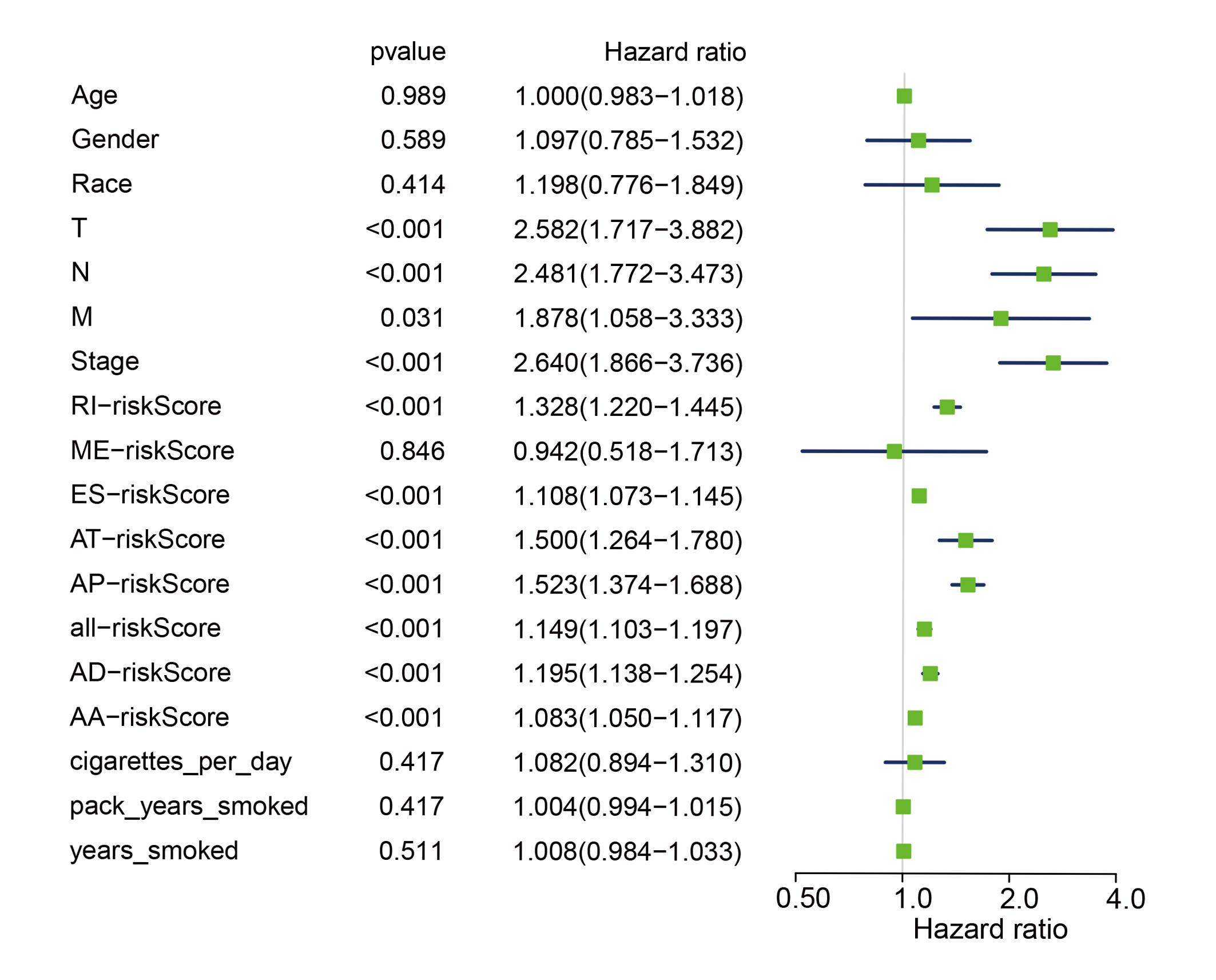

Supplement: Supplementary file 14 [file Image5.TIF]
