# Supplementary material for: Analysis of Genome-Wide Alternative Splicing Profiling and Development of Potential Drugs in Lung Adenocarcinoma
Source: Front Genet. 2021 Oct 19;12:767259. doi: 10.3389/fgene.2021.767259 (PMC8560713; doi:10.3389/fgene.2021.767259)
Supplement: Supplementary file 7 [file Table2.DOCX]

Table 2. The IC50 values of AZ-628 in LUAD

| Cell lines | IC50 (μM) | Pathology |
| --- | --- | --- |
| EKVX | 46.073 | primary |
| NCI-H1666 | 22.021 | metastasis |
| NCI-H1838 | 14.684 | primary |
| NCI-H322M | 13.661 | primary |
| NCI-H1355 | 8.016 | metastasis |
| LXF-289 | 6.070 | primary |
| NCI-H1975 | 4.781 | primary |
| NCI-H23 | 3.620 | primary |
| HOP-62 | 3.459 | primary |
| NCI-H1648 | 0.618 | metastasis |
| Calu-6 | 0.050 | primary |
