# Supplementary material for: Analysis of Genome-Wide Alternative Splicing Profiling and Development of Potential Drugs in Lung Adenocarcinoma
Source: Front Genet. 2021 Oct 19;12:767259. doi: 10.3389/fgene.2021.767259 (PMC8560713; doi:10.3389/fgene.2021.767259)
Supplement: Supplementary file 8 [file Table5.DOCX]

Table S2. The HR and *P* value of LUAD-all-multiCox

| Gene | Coefficient | HR | *P* value | Type |
| --- | --- | --- | --- | --- |
| SEC14L2 | -18.4713 | 9.51e-09 | 0.0001 | ES |
| MORF4L2 | 3.1356 | 23.0032 | 0.0023 | ES |
| CHEK2 | -16.3481 | 7.95e-08 | 0.0024 | ES |
| TRAPPC8 | -15.0823 | 2.82e-07 | 0.0005 | AA |
| IMMP1L | 0.9022 | 2.4650 | 0.0097 | ES |
| NTPCR | 3.5532 | 34.9262 | 0.0018 | AT |
| C10orf32 | -3.7913 | 0.0225 | 0.0238 | RI |
| EMCN | -7.8087 | 0.0004 | 0.0003 | ES |
| TTC39C | 0.5558 | 1.7434 | 0.0303 | AP |
| TMEM51 | 1.2848 | 3.6140 | 0.0291 | ES |
| MRPL33 | 0.4353 | 1.5455 | 0.0066 | ES |
| RAMP1 | 0.5469 | 1.7279 | 0.0366 | AP |
| ATP2A3 | -1.9084 | 0.1483 | 0.0113 | ES |
| LDB1 | -0.1383 | 0.8708 | 0.0182 | AP |
| KDM5A | 1.0075 | 2.7388 | 0.0364 | AT |
| FAM71D | 1.2197 | 3.3864 | 0.0078 | AT |
| GOLGA8M | -1.4166 | 0.2425 | 0.0117 | RI |

HR, hazard ratio.
