# Supplementary material for: Analysis of Genome-Wide Alternative Splicing Profiling and Development of Potential Drugs in Lung Adenocarcinoma
Source: Front Genet. 2021 Oct 19;12:767259. doi: 10.3389/fgene.2021.767259 (PMC8560713; doi:10.3389/fgene.2021.767259)
Supplement: Supplementary file 10 [file Table3.DOCX]

Table 3. The IC50 values of NVP-AUY922 in LUAD

| Cell lines | IC50 (μM) | Pathology | Cell lines | IC50 (μM) | Pathology |
| --- | --- | --- | --- | --- | --- |
| NCI-H2291 | 10.222535 | metastasis | HCC-78 | 0.032157 | metastasis |
| NCI-H1838 | 5.874921 | primary | ABC-1 | 0.031265 | primary |
| NCI-H322M | 5.214812 | primary | NCI-H1437 | 0.027666 | metastasis |
| NCI-H1563 | 4.243296 | primary | NCI-H2087 | 0.026917 | metastasis |
| NCI-H1666 | 3.44141 | metastasis | NCI-H358 | 0.024135 | primary |
| NCI-H1623 | 3.134648 | metastasis | NCI-H1651 | 0.023077 | primary |
| NCI-H1793 | 1.336631 | primary | PC-14 | 0.022175 | primary |
| EKVX | 0.237469 | primary | NCI-H2085 | 0.021596 | primary |
| NCI-H1435 | 0.198671 | primary | NCI-H1944 | 0.021242 | metastasis |
| NCI-H1573 | 0.180681 | metastasis | NCI-H1355 | 0.019943 | metastasis |
| NCI-H838 | 0.163924 | metastasis | LXF-289 | 0.01852 | primary |
| NCI-H1755 | 0.14273 | metastasis | NCI-H522 | 0.017519 | primary |
| NCI-H23 | 0.136121 | primary | NCI-H1568 | 0.016541 | metastasis |
| NCI-H441 | 0.09115 | metastasis | COR-L105 | 0.015269 | primary |
| NCI-H1781 | 0.088218 | metastasis | NCI-H1648 | 0.015064 | metastasis |
| Calu-6 | 0.079931 | primary | NCI-H2405 | 0.014614 | metastasis |
| RERF-LC-MS | 0.078131 | primary | NCI-H2228 | 0.013568 | primary |
| HCC-827 | 0.067775 | primary | NCI-H2009 | 0.01356 | metastasis |
| NCI-H2347 | 0.062501 | primary | NCI-H650 | 0.013443 | metastasis |
| RERF-LC-KJ | 0.061536 | primary | SK-LU-1 | 0.012373 | primary |
| NCI-H2023 | 0.053233 | metastasis | NCI-H2342 | 0.011881 | primary |
| SW1573 | 0.052505 | primary | HOP-62 | 0.010625 | primary |
| Calu-3 | 0.045172 | metastasis | NCI-H2122 | 0.010496 | metastasis |
| NCI-H1693 | 0.044356 | metastasis | NCI-H2030 | 0.009935 | metastasis |
| NCI-H1734 | 0.040819 | primary | NCI-H1975 | 0.008744 | primary |
| NCI-H1792 | 0.03453 | metastasis | HCC-44 | 0.008046 | primary |
