# Supplementary material for: Analysis of Genome-Wide Alternative Splicing Profiling and Development of Potential Drugs in Lung Adenocarcinoma
Source: Front Genet. 2021 Oct 19;12:767259. doi: 10.3389/fgene.2021.767259 (PMC8560713; doi:10.3389/fgene.2021.767259)
Supplement: Supplementary file 11 [file Table4.DOCX]

Table S1. Clinical characteristics of LUAD in TCGA data set

| characteristic | Entire series (%) |
| --- | --- |
| Gender |  |
| Male | 198/ 444(44.59) |
| Female | 246/444 (55.4) |
| Age(years) |  |
| >65 | 227/444(51.1) |
| ≤65 | 217/444(48.9) |
| Stage |  |
| I | 246/444 (55.4) |
| II | 109/444 (24.5) |
| III | 68/444(15.37) |
| IV | 21/444(4.73) |
| Race |  |
| Non-white | 59/444(13.3) |
| White | 385/444(86.7) |
| T stage |  |
| T1 | 164/444 (36.9) |
| T2 | 225/444 (50.7) |
| T3 | 39/444 (8.7) |
| T4 | 16/440 (3.7) |
| N stage |  |
| N0 | 293/444 (65.9) |
| N1 | 81/444(18.3) |
| N2 | 60/444(13.5) |
| N3 | 10/444(2.3) |
| M stage |  |
| M0 | 288/444(64.9) |
| M1 | 156/444(35.1) |
