# Supplementary material for: Analysis of Genome-Wide Alternative Splicing Profiling and Development of Potential Drugs in Lung Adenocarcinoma
Source: Front Genet. 2021 Oct 19;12:767259. doi: 10.3389/fgene.2021.767259 (PMC8560713; doi:10.3389/fgene.2021.767259)
Supplement: Supplementary file 12 [file Table6.DOCX]

Table S4. The HR and *P* value of LUAD-splicing factors

| Gene | HR | *P* value | Gene | HR | *P* value |
| --- | --- | --- | --- | --- | --- |
| SNW1 | 15.8094 | 0.0003 | DDX23 | 3.7648 | 0.0289 |
| RBM8A | 6.2688 | 0.0020 | INTS3 | 2.0976 | 0.0304 |
| RNF34 | 3.3133 | 0.0045 | DHX9 | 4.1211 | 0.0306 |
| TFIP11 | 3.2800 | 0.0124 | HNRNPM | 4.0627 | 0.0313 |
| PRPF3 | 2.3649 | 0.0154 | ZNF207 | 2.8882 | 0.0317 |
| PRMT5 | 2.5049 | 0.0160 | NCBP1 | 2.3443 | 0.0318 |
| MOV10 | 2.3960 | 0.0161 | ZCCHC8 | 2.4713 | 0.0325 |
| RNF213 | 1.8652 | 0.0188 | HNRNPR | 4.0627 | 0.0313 |
| DHX36 | 2.4142 | 0.0189 | DDX46 | 2.5696 | 0.0358 |
| HNRNPK | 19.7718 | 0.0203 | RBM25 | 2.1087 | 0.0367 |
| U2SURP | 2.3528 | 0.0207 | HNRNPU | 4.3425 | 0.0376 |
| SSB | 3.4860 | 0.0211 | NONO | 3.9497 | 0.0380 |
| INTS4 | 2.4501 | 0.0229 | CDK12 | 1.9670 | 0.0390 |
| HNRNPH1 | 2.7368 | 0.0236 | RBM45 | 2.8287 | 0.0391 |
| ZC3HAV1 | 2.4928 | 0.0259 | IGF2BP3 | 1.2143 | 0.0441 |

HR, hazard ratio.
